# One Step Ahead: The Perceived Kinematics of Others’ Actions Are Biased Toward Expected Goals

**DOI:** 10.1037/xge0000126

**Published:** 2015-11-23

**Authors:** Matthew Hudson, Toby Nicholson, William A. Simpson, Rob Ellis, Patric Bach

**Affiliations:** 1 Department of Psychology, University of Plymouth

**Keywords:** representational momentum, action prediction, predictive coding, mirror neurons, prediction error

## Abstract

Action observation is often conceptualized in a bottom-up manner, where sensory information activates conceptual (or motor) representations. In contrast, here we show that expectations about an actor’s goal have a top-down predictive effect on action perception, biasing it toward these goals. In 3 experiments, participants observed hands reach for or withdraw from objects and judged whether a probe stimulus corresponded to the hand’s final position. Before action onset, participants generated action expectations on the basis of either object types (safe or painful, Experiments 1 and 2) or abstract color cues (Experiment 3). Participants more readily mistook probes displaced in a predicted position (relative to unpredicted positions) for the hand’s final position, and this predictive bias was larger when the movement and expectation were aligned. These effects were evident for low-level movement and high-level goal expectancies. Expectations bias action observation toward the predicted goals. These results challenge current bottom-up views and support recent predictive models of action observation.

Action observation lies at the heart of social interaction. It allows people to infer others’ internal states, predict what they are going to do next, and coordinate joint actions (Hamilton & Grafton, 2007; Sebanz & Knoblich, 2009). Such abilities are typically explained in a bottom-up manner, where observed actions are matched to an action in the observers’ motor repertoire (Rizzolatti, & Sinigaglia, 2010). Recent findings, however, have challenged these views, revealing striking top-down effects on action observation, affecting the action’s neural encoding (de la Rosa, Streuber, Giese, Bulthoff, & Curio, 2014), one’s gaze response (Joyce, Schenke, Bayliss, & Bach, 2015; Teufel, Fletcher, & Davis, 2010), and the tendency to imitate (Bach, Bayliss & Tipper, 2011; Longo & Bertenthal, 2009).

Such effects have prompted the proposal that action observation, like perception in general, is inherently predictive and happens relative to top-down expectations (Bach, Nicholson & Hudson, 2014; Csibra, 2007; Kilner, 2011). In these views, originally developed to provide neuronally plausible computational models for motor control and low-level vision, the brain constantly makes predictions about forthcoming events on the basis of prior knowledge about the world and other people (Clark, 2013; Friston & Kiebel, 2009; Schütz-Bosbach & Prinz, 2007). These predictions are not abstract but are seamlessly integrated—in a process akin to visuomotor imagery (Vogt, Di Rienzo, Collet, Collins, & Guillot, 2013)—with sensory input and have direct perceptual consequences. Stimulation that matches the predictions is processed fluently and becomes biased toward the predictions. Prediction errors, however, highlight the unexpected event and allow one’s predictions—or the internal models from which they were derived—to be reevaluated (Clark, 2013; Friston & Kiebel, 2009).

One example of such predictions concerns motion perception. People perceive moving objects not where they currently are but displaced slightly into the future. For example, observers typically (mis-)perceive probe stimuli further along the predicted trajectory as identical with a moving object’s last seen position, while equal displacements in the opposite direction are readily detected (i.e., representational momentum; Freyd & Finke, 1984; for a review see Hubbard, 2005). These forward displacements reveal the expected pattern of predicted stimulation being integrated with perception, while mismatching events are enhanced. Moreover, in line with prediction models, they integrate both bottom-up sensory information (e.g., motion speed) and top-down expectations (e.g., naive physics) and emerge from low-level motion sensitive regions in the brain (Senior, Ward & David, 2002).

Can the perception of others’ actions be accounted for in such models? We test a core tenet of such a view, namely that the perception of even low-level features of others’ actions would similarly not only reflect bottom-up sensory input but would be biased by top-down expectations about others’ forthcoming actions (cf. Bach et al., 2014; Kilner, 2011). Such findings would be a marked departure from prior work, where kinematic information activates associated action goals, instead revealing the reverse influence of goals directly affecting action perception.

We adapted the classical representational momentum paradigm, originally devised to study nonsocial motion prediction processes, to test this notion. Participants watched actors either reaching toward or withdrawing from objects that were safe (e.g., wine glass) or painful to touch (e.g., cactus). To ensure that participants would form an expectation about the forthcoming actions, participants instructed the actor about the appropriate action with the object. When the object was safe, participants instructed a reach (e.g., saying “Take it”), and when it was painful, a withdrawal (e.g., “Leave it”). Because such imperative language cues elicit visuomotor imagery (Glenberg & Kaschak, 2002) and are used to guide others’ behavior (Wolpert, Doya, & Kawato, 2003), they should create strong action expectations. As soon as participants made these statements, the hand either reached for the object or withdrew from it, conforming to or violating the expectation. Midway during the movement the hand disappeared. Participants judged whether a probe stimulus in the just-seen final position, slightly further along the trajectory (predicted position), or slightly behind (unpredicted position) was either the same or different from the hand’s final position.

Biological motion elicits predictive displacements that are similar to those for nonbiological motion (Hudson, Burnett, & Jellema, 2012; Hudson & Jellema, 2011; Hudson, Liu, & Jellema, 2009; Thornton & Hayes, 2004; Wilson, Lancaster, & Emmorey, 2010), and the characteristic kinematics enable observers to mentally simulate even complex action trajectories when they become occluded from view (Cross, Stadler, Parkinson, Schutz-Bosbach, & Prinz, 2013; Parkinson, Springer, & Prinz, 2012; Stadler et al., 2011). Here, we test a key assumption of hierarchical feedback models of social perception: that high-level expectations about others’ forthcoming actions directly feed into these predictions and selectively bias the perception of these actions toward these goals. Expectations to reach (saying “Take it”) should then increase perceptual shifts toward the object, whereas expectations to withdraw (“Leave it”) should lead to shifts away from the object. Across three experiments, we varied whether these expectancies referred to action kinematics (Experiment 1), action goals (Experiment 2), or participants’ verbal statements based not on object type (painful/safe) but on object color (randomly allocated), which allowed us to investigate their “pure” influence, independent of object painfulness (Experiment 3).

## Method

### Participants

Participants (Experiment 1: *N* = 46, Experiment 2: *N* = 42, Experiment 3: *N* = 36) were right-handed, had normal or corrected-to-normal vision, and were native English speakers. They gave written informed consent and received course credit or £6 (US$9.25) for their participation. Of these, only those who could distinguish visually between the experimentally manipulated probes in a training session (see the Procedure section) progressed to the main experiment (Experiment 1: *N* = 40, 26 females, mean age = 23.3 years, *SD* = 8.9; Experiment 2: *N* = 32, 22 females, mean age = 23.3 years, *SD* = 6.9; Experiment 3: *N* = 36, 28 females, mean age = 21.6 years, *SD* = 6.0 years). Participants were further screened on the basis of catch trial performance during the experiment (see the Exclusion Criteria section). This two-stage exclusion process was selected a priori and, because this was the first demonstration of the effect and random response strategies are high in difficult feedback-less visual tasks such as ours (DeRight & Jorgensen, 2015), deliberately conservative. Data collection stopped if the experimenter was confident that, after exclusion, a minimum sample size off 22 would be surpassed in each experiment (on the basis of a power analysis of a previous study of the effect of an actor’s eye gaze on representational momentum; Hudson et al., 2009).

### Apparatus and Stimuli

**Figure 1 fig1:**
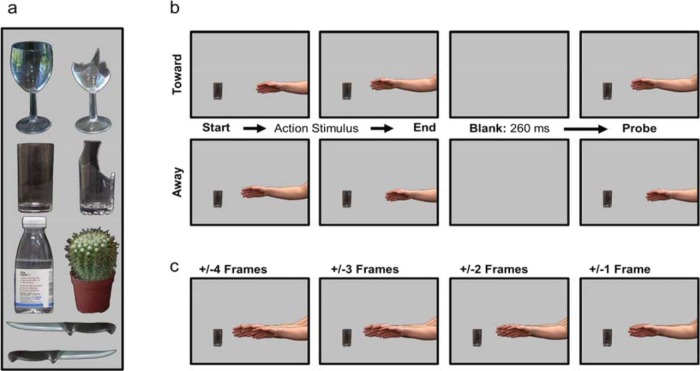
Experimental stimuli. The safe objects (Panel a, left column) and the paired dangerous objects (Panel a, right column), and the knife oriented safely or dangerously with respect to the hand (Panel a, bottom). The trial sequence of a reach toward (Panel b, top) or withdrawal from (Panel b, bottom) an object (action stimulus), followed by a blank screen and then the probe stimulus. In these examples, the probe position is the same as the final action stimulus frame. The probe stimulus levels are depicted in Panel c. In each image, the center hand is the same as the one in the final action stimulus frame in Panel b (top), and the different probe stimuli are superimposed on either side of it. For reaches toward the object, the probe nearest the object was the predicted probe and the probe farthest from the object was the unpredicted probe. For reaches away from the object, the probe farthest from the object was the predicted probe and the probe nearest the object was the unpredicted probe. The difference between the same and different probes decreases across the images from left to right (4 frames, 3 frames, 2 frames, 1 frame). For illustrative purposes, the background is depicted in gray instead of black. See the online article for the color version of this figure.

Videos of an arm reaching for one of four safe-to-grasp objects (see the left half of Figure 1a) were filmed at 30 fps with a Canon Legria HFS200 and were digitally manipulated using MovieDek and Corel Paintshop Pro ×6. Background details were replaced with a uniform black background. A second set of stimuli was created by digitally replacing the object with a painful object of similar size and shape (see the right half of Figure 1a), resulting in four additional action sequences matched for reach trajectory. In Experiment 3, stimuli were modified by placing a green or red overlay of 30% opacity over the objects, giving them an either red or green tint.[Fig-anchor fig1]

The first 26 frames of each video depicting the reaching portion of the action were used for the action sequences and probe stimuli. Each action sequence consisted of 3, 4, or 5 frames. The first frame was randomly selected between Frames 13 and 17. The action proceeded in two frame jumps by adding or subtracting 2 to the frame number for reaches (e.g., 13–15–17) and withdrawals (e.g., 13–11–9), respectively. The duration of each frame was 80 ms, presenting the action in near-real time. Stimuli occupied an area of .07° × .12°, given a viewing distance of 60 cm.

The experiment was performed using Presentation (16.3; 2012) software on a Viglen DQ67SW computer and Philips Brilliance 221P3LPY monitor (resolution: 1920 × 1080, refresh rate: 60 Hz). Participants wore Logitech PC120 headphones and microphones.

### Procedure

Each trial began with the first frame of the movie as a static image, showing the hand in a neutral position and an object. Participants said “Forward” for safe objects and “Backward” for painful objects in Experiment 1, “Take it” and “Leave it” in Experiment 2, and “Forward” for green objects and “Backward” for red objects in Experiment 3. The action sequence started 1,000 ms after word onset (detected via Presentation’s sound threshold logic). The probe followed the action sequence after a blank of 260 ms (see Figure 1b), showing the hand either in the same position as in the final frame of the action sequence (same probe), further along the trajectory (predicted probe), or in a position just prior to the final frame (unpredicted probe). Participants pressed the space bar if they thought the probe hand’s position was different from the hand’s final position and did nothing if they thought it was identical.

#### Training procedure

Participants first completed four training blocks (36 trials each), in which no verbal response was required, to familiarize themselves with the task and allow us to assess their ability to distinguish the probes. In the first block, the probe differed from the final position by four frames (±4, sufficiently obvious for the task to be learned with ease) and decreased by one frame in each block (±3, ±2, ±1; see Figure 1c), such that task difficulty progressively increased.

Participants’ overall accuracy (average of correct responses across all probe types) and sensitivity (proportion of same response for same probes compared with same response for different probes) were used to assess performance. Below-chance performance on either measure (accuracy, 50%; sensitivity, 0%) was considered inadequate. If the participant passed the ±1 block, the experimental probe was set at ±1 frame (Experiment 1: *n* = 28; Experiment 2: *n* = 25; Experiment 3: *n* = 26). If the participant did not pass the ±1 block but passed the ±2 block, the experimental probe was ±2 frames (Experiment 1: *n* = 12; Experiment 2: *n* = 7; Experiment 3: *n* = 10). Failure to pass either block meant they did not proceed to the experimental session (Experiment 1: *n* = 6; Experiment 2: *n* = 10; Experiment 3: *n* = 0).

#### Experimental procedure

Each experiment presented four iterations of Object (painful, safe) × Action (reach, withdrawal) × Movie Length (3, 4, 5 frames) × Probe (predicted, same, unpredicted), producing 144 trials. In Experiment 3, object color was randomly selected on each trial. The distribution of red and green objects across the levels of action direction, object type, and probe did not differ significantly from chance, χ^2^(11) = 3.73, *p* = .98. Frames were positioned on the horizontal midline, but varied along the *x*-axis across trials. Twenty-four catch trials in which the probes were ±4 frames from the final position were randomly interspersed. These trials with obvious displacements identified participants who disengaged with the task and responded randomly. Three breaks were provided at 56-trial intervals.

### Analysis

Representational momentum was measured as the difference between the frequency of detected unpredicted probes relative to predicted probes. Positive numbers therefore reflect increased likelihoods of accepting predicted displaced hands as “same” compared to unpredicted displacements (the representational momentum effect). This measure corresponds to the original approach (Freyd, & Finke, 1984) and allows straightforward measurement of perceived forward displacements without further assumptions. Responding to only probes perceived as different links responses to the “prediction error” elicited by the probes and eliminates confounding influences such as spatial compatibility effects elicited by multiple response keys.

### Exclusion Criteria

The same exclusion criteria were applied in all experiments. Responses faster than 200 ms or slower than 3,000 ms were excluded (Experiment 1: 0.3%, Experiment 2: 1.4%, Experiment 3: 1.1%). Participants were excluded if catch trial performance was below 1 *SD* of the group mean accuracy or did not reveal at least a minimal improvement (>10% more detections) over the experimental trials (Experiment 1: *n* = 9; Experiment 2: *n* = 8; Experiment 3: *n* = 5).

## Results

Data were collected as three separate experiments, but for brevity and methodological similarity, we present them as a single analysis with experiment as a between-subjects variable but with separate analyses for each experiment to demonstrate the robustness of the effects.

**Figure 2 fig2:**
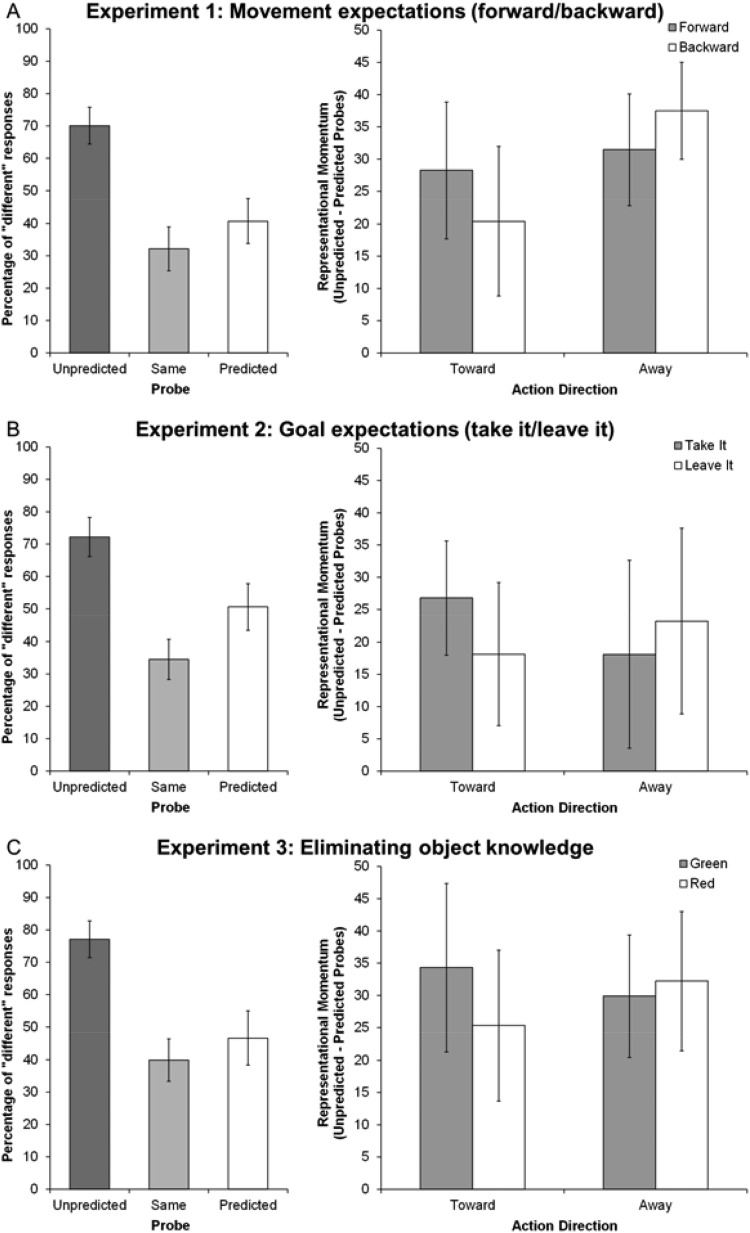
The representational momentum effect and the effect of prior expectation. Each row of graphs represents a different verbal response made prior to action onset. Participants said “Forward” if the object was safe and “Backward” if the object was dangerous (Panel A). Participants said “Take it” if the object was safe and “Leave it” if the object was dangerous (Panel B). The color of the object was randomly assigned as red or green, independent of object type, and participants said “Forward” if the object was green and “Backward” if the object was red (Panel C). The left column in each panel depicts the proportion of responses in which participants judged the position of the probe stimulus to be different from the final position of the action stimulus, for the three different types of probe. The right column depicts the interaction between prior expectation and action direction on the size of the representational momentum effect (unpredicted probe detections compared with predicted probe detections). Error bars represent 95% confidence intervals.

We first established the presence of representational momentum by entering the proportion of “different” responses into an analysis of variance ANOVA with the within-subject variable probe (predicted, same, unpredicted) and the between-subjects variable experiment. There was a main effect of probe, *F*(2, 166) = 156.3, *p* < .001, η_p_^2^ = .653, with more “different” responses for unpredicted displacements than predicted displacements, *t*(85) = 9.96, *p* < .001, *d* = 1.5, 95% confidence interval (CI) [23, 33], confirming the classic representational momentum effect. Fewer displacements were (erroneously) reported for same probes than for predicted probes, *t*(85) = 5.14, *p* = .004, *d* = 0.51, 95% CI [6, 14], and unpredicted probes, *t*(85) = 18.6, *p* < .001, *d* = 2.23, 95% CI [34, 42]. There was no effect of experiment (*p* = .134) and no interaction (*p* = .395). The effect of probe was present in each experiment, *F*(2, 60) > 39.0, *p* < .001, η_p_^2^ > .596 for all (see Figure 2, left column).[Fig-anchor fig2]

To test whether prior expectancies affect representational momentum, we entered the size of the representational momentum effect (unpredicted probe detections minus predicted probe detections) into an ANOVA with the within-subject variables expectancy (reach, withdrawal) and action (reach, withdrawal) and the between-subjects variable experiment. This analysis revealed only the predicted Expectancy × Action interaction, *F*(1, 83) = 14.98, *p* < .001, η_p_^2^ = .153, 95% CI [7, 20], observed power = .95, and no other effects (all *F*s < 1.17, *p* > .291). As predicted, representational momentum was greater when expectancy and action matched than when they mismatched, for both reaches, *t*(85) = 2.44, *p* = .017, *d* = 0.17, 95% CI [1, 9], and withdrawals, *t*(85) = 3.08, *p* = .003, *d* = 0.28, 95% CI [3, 14]. This interaction was evident for all experimental groups when analyzed separately (see Figure 2, right column), Experiment 1: *F*(1, 30) = 4.89, *p* = .035, η_p_^2^ = .140, 95% CI [2, 26], observed power = .89; Experiment 2: *F*(1, 23) = 4.67, *p* = .041, η_p_^2^ = .175, 95% CI [1, 26], observed power = .90; Experiment 3: *F*(1, 30) = 4.31, *p* = .047, η_p_^2^ = .126, 95% CI [1, 22], observed power = .73.

Further experiments (see the online supplemental materials) replicated the effects with other instruction statements (e.g., “Stop”/“Go”), *F* = 12.37, *p* = 001, but not when participants merely identified object painfulness (saying “Painful” or “Safe”), named the objects (e.g., “Glass” vs. “Cactus”), or made equivalent nonverbal manual actions (pushing a joystick forward/backward instead of saying “Forward”/“Backward”) that similarly matched or contradicted the actors’ movement but which crucially would not be expected to dictate the goal of the actors’ behavior.

## Discussion

When judging the disappearance point of a moving hand, participants more readily (mis-)identified probes further along the trajectory as the hands’ final position than probes in an unpredicted position. This is in line with the idea that expectations about forthcoming motion are integrated, in a Bayesian manner, with actual perception, such that stimuli were perceived further along the trajectory than they actually were (Roach, McGraw & Johnston, 2011). Our study now reveals that, for the perception of others’ actions, this predictive bias is driven by prior expectations about the forthcoming action. Instructing the actor to reach for the object (“Forward,” “Take it”) lead to a stronger perceptual displacement toward the object, whereas instructing a goal to withdraw (“Backward,” “Leave it”) increased displacements away from it. These effects were observed not only when the expectations concerned movement kinematics (Experiment 1) but also when those kinematics were implied by an action goal that required those movements to achieve it (Experiment 2), even when the instructions were not meaningfully related to object type (Experiment 3).

Further experiments (see the online supplemental material) confirmed that the effects could not be explained through implicit or explicit processing of the objects’ affordances (painful or safe) or through nonspecific activation of forward and backward codes (pushing a joystick forward and backward instead of saying “Forward” or “Backward”). The perceptual displacements of observed actions therefore reflect specifically one’s expectations to be able to set a goal for the other person’s action, rather than more unspecific influences of conceptual or motor processes. These data therefore reveal, for the first time, that top-down expectations about others’ actions directly bias perceptual judgments of the predicted action toward the anticipated goals. Moreover, because participants were instructed to report displacements accurately, the data reveal involuntary influences of action expectations on judgments on action kinematics.

Such effects are hard to account for in conventional models, which conceptualize action observation as a bottom-up process that matches, on the basis of kinematic information, observed actions to action goals (di Pellegrino, Fadiga, Fogassi, Gallese, & Rizzolatti, 1992; Iacoboni, 2009; Rizzolatti, & Sinigaglia, 2010). Instead, they support recent predictive models of social perception (Bach et al., 2014; Csibra, 2007; Kilner, Friston, & Frith, 2007b) in which any high-level expectation about others’ behavior is immediately translated into concrete predictions of their forthcoming actions (Vogt et al., 2013; Kilner, Friston, & Frith, 2007a). These predictions are integrated with incoming sensory information, biasing them into the future. In contrast, mismatches elicit salient prediction errors and reevaluations of prior expectations (Csibra, 2007; Neal & Kilner, 2010).

Such hierarchical models provide a step change in the understanding of social perception. First, they offer a unifying basis for predictive effects in social perception, such as people’s remarkable ability for maintaining a dynamic update of complex actions should they become occluded from view (Parkinson et al., 2012; Springer, Brandstädter, & Prinz, 2013) and the ability to coordinate their own actions with others’ future behavior (Wilson & Knoblich, 2005). Moreover, the models point toward a common framework under which research on social and nonsocial perception (Guo et al., 2007; Summerfield et al., 2006), as well as motor control and computational neuroscience, can be integrated (cf. Clark, 2013). Note, for example, that in prior nonsocial perception research, top-down expectations were induced by manipulating variables such as contextual motion cues or presentation frequency. In contrast, in our studies, the expectations either reflected action goals (“take it”/“leave it”) or were spatially meaningful only from the perspective of the observed actor, not the participant (“forward”/“backward”). That these manipulations nevertheless induced perceptual displacements suggests that even core processes of social perception such as goal understanding and visuospatial perspective taking can be seamlessly integrated into one’s models of the other person and drive subsequent perception and action.

Two aspects need to be addressed by further studies. First, the instruction task used here required participants to explicitly generate expectations about forthcoming actions. This differs from social situations in which others’ behavior is self-determined and expectations are inferred from social cues. Thus, although our data reveal that action expectations automatically cascade downward and affect the perception of subsequent behavior, it is important to delineate under what circumstances, and from what cues, such expectations are derived during everyday social interactions (e.g., the actor’s own statements; see Hudson, Nicholson, Ellis, & Bach, in press). A second question concerns the role of visuomotor imagery. The generation of expected sensory stimulation is a crucial part of top-down prediction models, and visuomotor imagery or simulation processes have been proposed as a means by which high-level expectations can be integrated with the current context (Kilner et al., 2007b, 2007a; Vogt et al., 2013; Wilson & Knoblich, 2005). Although our data show that action expectations indeed have such a perceptual component, future studies need to test further assumptions of prediction models, for example that the weighting of these sensory expectations relative to the actual stimulation differs with the reliability of (a) the actor in following the verbal cues and (b) precision of the bottom-up motion signals.

## Supplementary Material

10.1037/xge0000126.supp
