# Supplementary material for: One Step Ahead: The Perceived Kinematics of Others’ Actions Are Biased Toward Expected Goals
Source: J Exp Psychol Gen. 2015 Nov 23;145(1):1–7. doi: 10.1037/xge0000126 (PMC4694084; doi:10.1037/xge0000126)
Supplement: Supplementary file 1 [file xge-XGE-2014-0717-Supplemental.docx]

**Supplementary Experiment 1**

**Action vs. object based expectancies**

To further explore the influence of prior expectations on action prediction, we conducted a further experiment with two new word pairs in a within-subjects design. The first word pair was dynamic (Go/Stop) and the second word pair related to the properties of the object (Harmless/Painful). These words afforded the opportunity to test, first, if the influence of the expectancies extended to features of the object itself, rather than only words related to the anticipated movement of the other person. This is important because painfulness itself, irrespective of whether participants focus on the action one performs with the object, could give rise to expectancies of withdrawals. In addition, the experiment allowed us to test whether the effects on representational momentum can also be evoked when the words said by the participant only imply withdrawals and reaches in the given context, but do not directly provide information about the direction of the forthcoming reach.

**Method**

**Participants.** Sixty participants took part in the experiment, but three did not pass the training session. The remaining 57 participants (39 females) had an average age of 26.3 years (*SD* = 10.3).

**Procedure.** All stimuli and apparatus are the same as for Experiment 1.

***Training session.*** Participants completed the same training trials as in Experiment 1, however the initial +/-4 training block was not given, and participants instead completed the remaining three blocks (+/-3, +/-2, +/-1).

***Experimental session.*** In one block, participants said “Go” if the object was safe and “Stop” if the object was dangerous. In another block, participants said “Harmless” if the object was safe and “Painful” if the object was dangerous. The order of the blocks was counterbalanced across participants. Each experimental block presented 2 iterations of object type (2) X motion direction (2) X movie length (3) X probe stimulus (3) producing 72 experimental trials in total, with an additional 12 catch trials. A break was provided half way through each block.

**Results**

The mean proportion of detected displacements in the catch trials was 91% (*SD* = 10%) and 53% (SD = 14%) in the experimental trials. Fifteen participants were excluded based on performance and 1.1% of trials were excluded based on reaction time.

We first ran a one-way ANOVA to establish the presence of the RM effect. As in the experiments reported in the paper, this analysis revealed a significant main effect of probe (*F*(2,82) = 101.7, *p* < .001, *η_p_^2^ =* .713). The proportion of detected displacements was higher for the unpredicted probes than the predicted probes (*t*(41) = 10.2, *p* < .001, *d* = 1.78, *95% CI* [25, 36]) and the same probes (*t*(41) = 14.2, *p* < .001, *d* = 1.9, *95% CI* [27, 36]), with no difference between the predicted and same probes (*t*(41) = .468, *p* = .642, *d* = .06, *95% CI* [-3, 5]).

The representational momentum effect (unpredicted – predicted) was entered into a three-way ANOVA with Expectation Type (dynamic vs. object property), Expectation (toward vs. away), and Action Direction (toward vs. away) as within-subjects factors. There was a significant interaction between Expectation and Action (*F*(1,41) = 12.4, *p* = .001, *η_p_^2^ =* .23, 95% CI [6, 20], observed power = .94, Supplementary Fig. 1). There were no further main effects or interactions, and there was no three-way interaction between Expectation Type, Expectation, and Action (*F*(1,41) = .514, *p* = .478, *η_p_^2^ =* .012, 95% CI [-13, 29]). However, a post-hoc analysis revealed that the two-way interaction between Expectation and Action was present only for the Go/Stop expectation type (*F*(1,41) = 7.49, *p* = .009, *η_p_^2^ =* .154, 95% CI [5, 28], observed power = .89), but did not reach significance for the Harmless/Painful word pair (*F*(1,41) = 1.6, *p* = .213, *η_p_^2^ =* .036, 95% CI [-5, 22], observed power = .33), even though, numerically, the effect was present in both conditions.

*
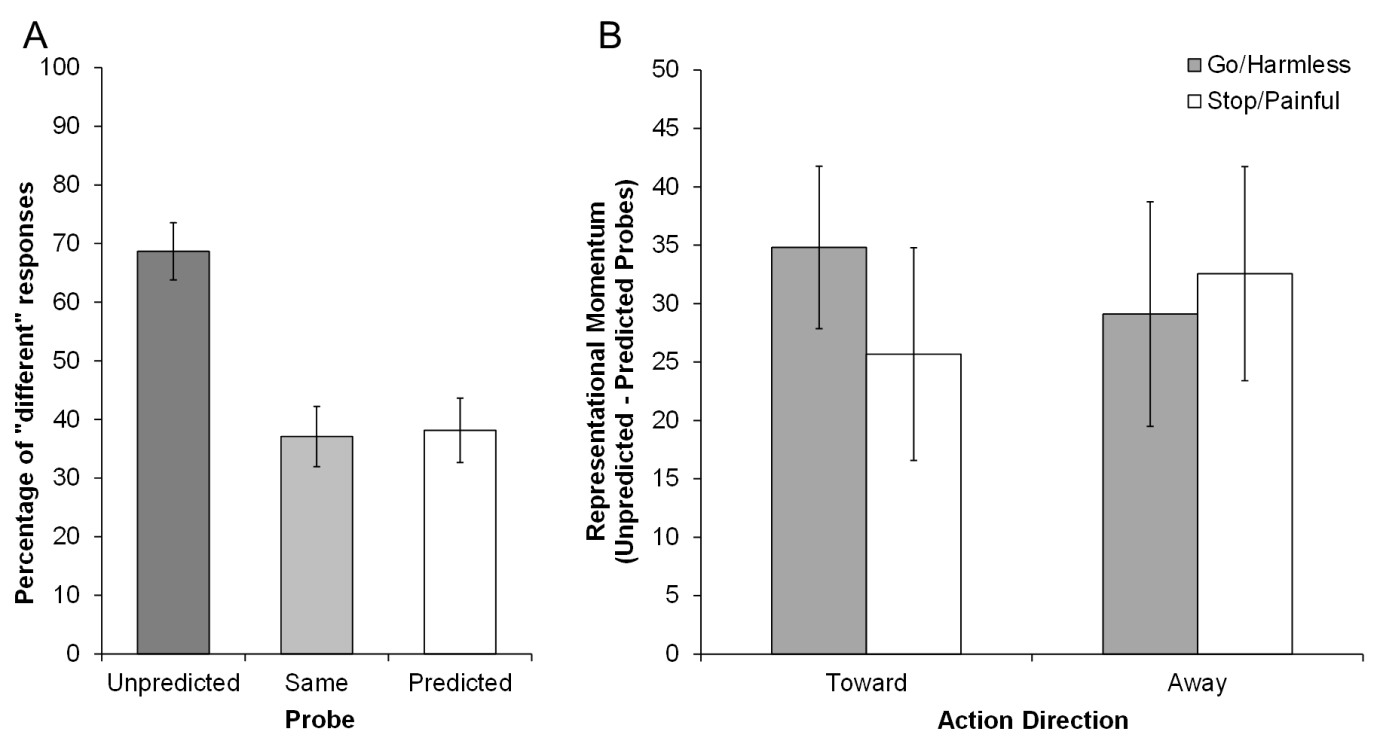
*

**Supplementary Fig. 1.**

The Representational Momentum effect and the effect of prior expectation. The proportion of reported displacements (a) when the probe was either the same as the final position of the action stimulus, or displaced in either a predicted or unpredicted position. The representational momentum effect (% “different” responses for unpredicted probes – predicted probes) (b) for each action direction and each object type after participants had said Go/Harmless for safe objects and Stop/Painful for dangerous objects. Error bars represent 95% confidence intervals.

**Discussion**

These results mirror those of the previous experiments and provide further evidence for the influence of prior expectations on the perception of subsequent movement. The size of the representational momentum effect was modulated depending on the congruency of a prior word with the direction of subsequent movement. A further finding of note is that the influence of word-action congruency on the representational momentum effect was evident most strongly for the words Go and Stop, and not for the words Harmless and Painful (although this differential effect was not supported statistically). It is therefore possible that the effect of expectations on action perception are most powerful when they are relevant to the action affordances elicited by the object rather than if they are tied to more general object properties.

**Supplementary Experiment 2**

**Effects of object identity**

The aim of this experiment was to investigate if expectations regarding the future movement of the actor are derived from the object itself. In this experiment, the verbal response made by participants simply related to the identity of the object and did not imply any action related affordances. An effect of congruency between object type and action on action prediction would suggest that expectancies are automatically generated from affordances derived from processing object identity. In contrast, if there is no effect of congruency, then this would imply that processing of object identity is insufficient to influence action prediction and that movement related affordances must be explicitly verbalised.

**Method**

**Participants and Procedure.** There were 34 participants (mean age = 23.0 years, *SD* = 8.9 years, 24 females). All participants passed the training session. Participants completed 3 training session blocks (+-3, +/-2, +/-1). In the experimental session participants completed the same 144 trials as in Experiment 1. The verbal response participants were required to make before onset of the action was to name what type of object it was (glass, wine glass, cactus, bottle, knife).

**Results**

The mean proportion of detected displacements in the catch trials was 97% (*SD* = 9%) and 58% (SD = 15%) in the experimental trials. Four participants were excluded based on performance and 1.8% of trials were excluded based on reaction time.

The one-way ANOVA revealed a significant effect of probe (*F*(2,58) = 56.9, *p* < .001, *η_p_^2^ =* .662). The representational momentum effect was highly significant (unpredicted vs. predicted: *t*(29) = 7.32, *p* < .001, *d* = 1.77, 95% CI [25, 44]). The proportion of reported displacements for the same probe was significantly less than for the unpredicted probe (*t*(29) = 11.4, *p* < .001, *d* = 2.01, 95% CI [29, 40]) but not the predicted probe (*t*(29) = .05, *p* = .96, *d* = .01, 95% CI [-6, 7]) .

The representational momentum effect was entered into a two-way ANOVA with Object Type (safe vs. dangerous) and Action (toward vs. away) as within-subjects factors. There were no main effects of Object Type or Action (all *F* < 1.13, all *p* > .296). Importantly, the two-way interaction was not significant (*F*(1,29) = .015, *p* = .904, *η_p_^2^ =* .000, 95% CI [-9, 10], observed power = .05, Supplementary Fig. 2).


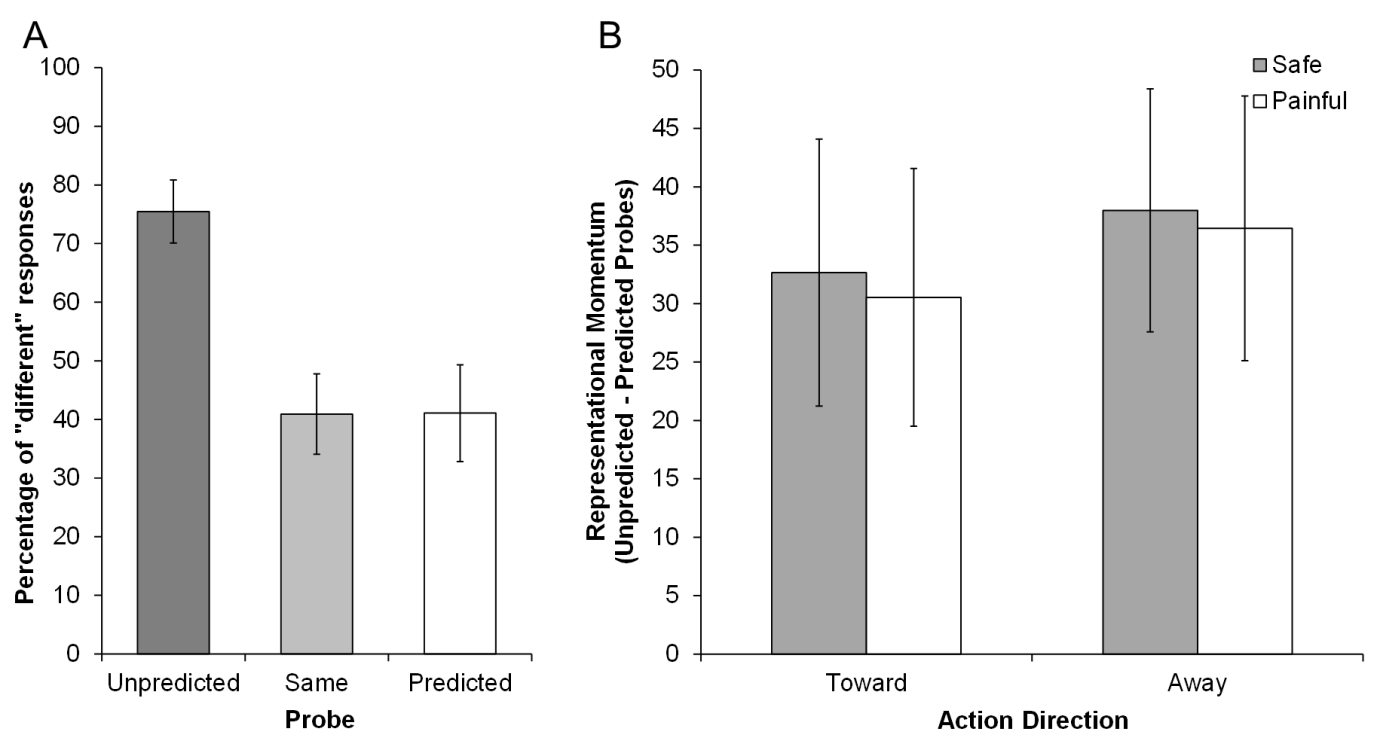


**Supplementary Fig. 2.**

The Representational Momentum effect and the effect of prior expectation. The proportion of reported displacements (a) when the probe was either the same as the final position of the action stimulus, or displaced in either a predicted or unpredicted position. The representational momentum effect (% “different” responses for unpredicted probes – predicted probes) (b) for each action direction and object type after participants had named the identity of the object. Error bars represent 95% confidence intervals.

**Discussion**

Although there was a significant representational momentum effect, this was not modulated by naming the object. That is, despite participants being required to process what the object is, the factor of whether the object was safe or painful to grasp did not create an expectancy of which direction the actor would move, and there was therefore no influence on subsequent action prediction. Taken in conjunction with the previous experiments, this suggests that action prediction is influenced by prior expectation of the actual kinematics of the action (what the action will look like) but that this does not necessarily require comprehension of the object itself.

**Supplementary Experiment 3:
Training session data across experiments**

The training session provides an opportunity to investigate the effect of object type in the absence of any explicit expectation. Previous studies have shown that aversive affordances are automatically elicited by the sight of dangerous objects (Anelli, Ranzini, Nicoletti & Borghi, 2013) or seeing someone else interact with them (Morrison, Peelen & Downing, 2007). Yet, the results presented in Supplementary Experiment 2 suggest that the object itself in the absence of an explicit verbal label to withdraw from the object is not sufficient to modify one´s prediction of another person´s action. However, it is possible that having participants focus on the identity of the object diverted attention from the affordances automatically derived from the object. In the training sessions, participants observed the same trial sequence and performed the same RM task as in the previous experiments, except no verbal response was made to the object itself. If action affordances are automatically derived from the type of object, then these will affect the observer´s expectation of the subsequent action in the same way as when participants had made an explicit action related word to the object. That is, there will be greater RM for reaches toward safe objects and reaches away from dangerous objects, than vice versa. Only those participants who were analysed in the experimental sessions were included in the analysis (*N* = 156), and the difficulty level (+/- 2, +/- 1) was that which participants completed in the experimental session.

The one-way ANOVA revealed a significant effect of probe (*F*(2,310) = 203.8, *p* < .001, *η_p_^2^ =* .568). The proportion of the detected displacements was significantly higher for the unpredicted than predicted probes (*t*(155) = 12.4, *p* < .001, *d* = 1.45, 95% CI [24, 33]), both of which were significantly greater than the reported displacements for the same probe (unpredicted: *t*(155) = 22.7, *p* < .001, *d* = 2.06, 95% CI [34, 41]; predicted: *t*(155) = 5.06, *p* < .001, *d* = .45, 95% CI [6, 13]).

The representational momentum effect was entered into a two-way ANOVA with Object Type and Action as within-subjects factors. There were no main effects of either Object Type or Action (all *F* < .73, all *p* > .394), and no two-way interaction (*F*(1,155) = 2.6, *p* = .109, *η_p_^2^ =* .016, 95% CI [-23, 2], observed power = .70, Supplementary Fig. 3). If anything, numerically the data showed the opposite pattern as that observed in the main experiments, with more RM whenever action and expectation mismatched, compared to when they matched.


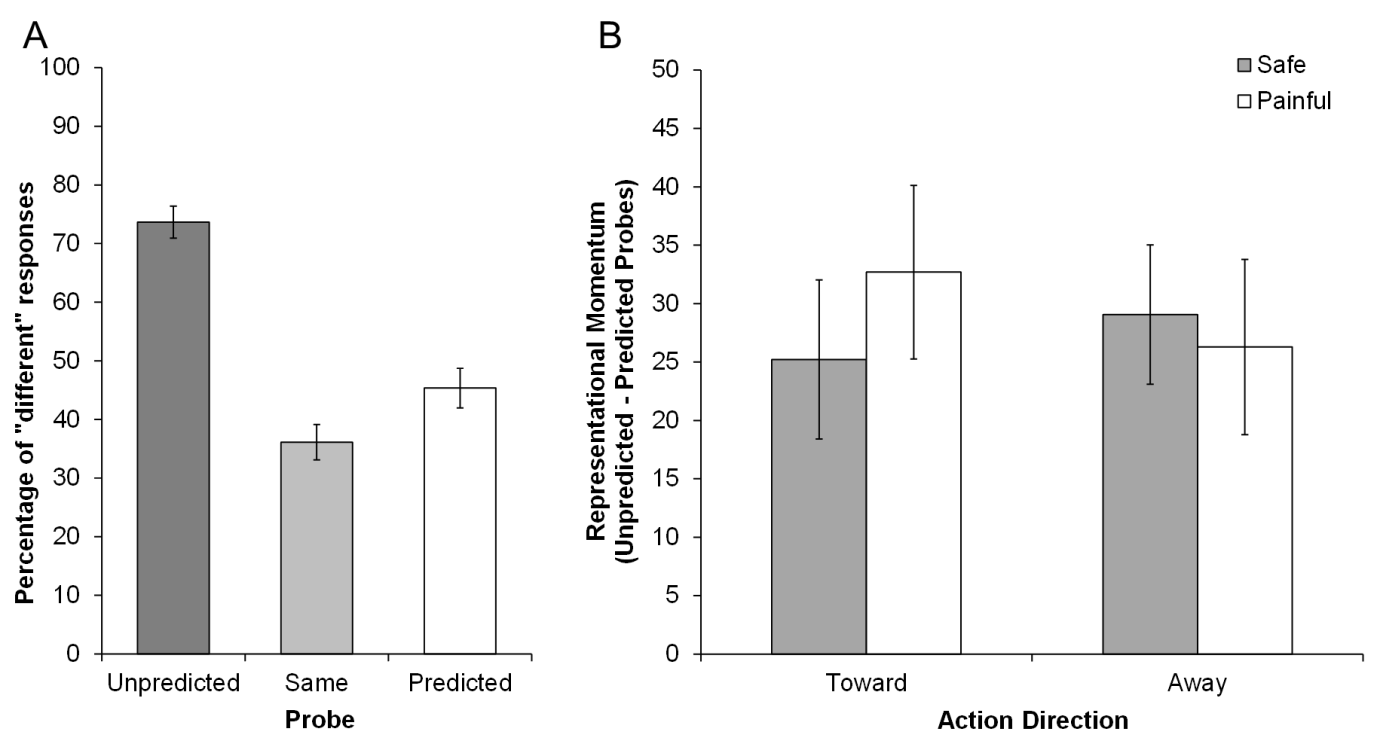


**Supplementary Fig. 3.**

The Representational Momentum effect and the effect of prior expectation. The proportion of reported displacements (a) when the probe was either the same as the final position of the action stimulus, or displaced in either a predicted or unpredicted position. The representational momentum effect (% “different” responses for unpredicted probes – predicted probes) (b) for each action direction and each object type in the absence of any prior verbal response to the object itself. Error bars represent 95% confidence intervals.

**Discussion**

Although the RM effect was highly significant, it was not affected by the congruency between the type of object and the direction of the arm reach. That is, expectations were not generated automatically from the object, despite previous research suggesting that painful objects automatically elicit an affordance to withdraw from them. This suggests that, neither explicit (Supplementary Experiment 2) nor implicit processing of the object itself is sufficient to influence the perception and prediction of another person’s subsequent actions, and that affordances only influence subsequent action prediction when they are explicitly verbalised. However, these results must be interpreted with caution, as in the training sessions there was no requirement to attend to the object, and so there is no guarantee that participants processed the object to the same degree as in the experimental sessions.

**Supplementary Experiment 4**

**Action execution has no effect on the prediction of others’ actions**

Here, we further verify our assumption that our effects arise because the instruction situation generates an expectation that the other person will comply with the participants’ verbal statements, and execute the instructed action. In other words, the instruction to “take it!” or “leave it!” is a means to induce a goal state in the other actor, which participants expect to directly guide the actor’s behaviour. If correct, then these effects should be eliminated if the action of the participants generates no such expectation that the actor will comply. We tested this by simply replacing the speech acts of the participants with other actions that typically do not trigger behaviour in others, but share all other relevant characteristics. Instead of verbally instructing the actor, we had participants execute a push or pull movement with a joystick. If the object was safe to grasp they pushed the joystick forward, and if the object was painful to grasp they pulled the joystick backwards.

These manual actions share all other components with the speech acts manipulated in the main experiments. In both setups, there is the requirement to retrieve the appropriate action for the object (forward or backward) and to actively use this knowledge for one’s own actions, one verbal and one manual. If the previous action prediction effects indeed reflect goals implied by the instruction situation, then we expect no influence of the manual actions on subsequent RM effects. If, however, the action prediction effects are a generalised non-social by-product, we would replicate the previous experiments and expect a larger action prediction effect for actions that are congruent with the executed action than incongruent.

To maximise the opportunity of obtaining an effect, we conducted two experiments, and varied the time interval between executed and observed action onsets. In the main experiments, the action began 1000 ms after the sound threshold had been reached to ensure that participants had sufficient time to complete the verbal response before the action began. In Experiment 1, we replicated this time interval, such that the offset between executed and observed action was 1200 ms. After finding no effect, we reduced the interval to 200 ms in Experiment 2 to reflect the time interval between offset of the verbal response and onset of the observed action, and to prevent any decay in the co-activation that may have occurred with a delay of 1200 ms. Again, no effect was found. As the results were the same and the designs were nearly identical, the two experiments will be described and analysed together, with Experiment (1200 ms, 200 ms) as an independent measures factor.

**Method**

*Participants*

Participants (Experiment 1: N = 26, Experiment 2: N = 25; mean age = 28 years, SD = 10.6, 36 females) were recruited from Plymouth University and wider community and took part in exchange for course credit or payment. All were native English speakers, right handed, and had normal/corrected vision.

*Stimuli and Apparatus*

A Thrustmaster USB joystick was used for participant’s action execution responses. JoytoKey software was used to read the joystick movements as a mouse movement, which was recorded by the experimental software Presentation. All other stimuli and apparatus were the same as in the main experiments.

*Procedure*

Participants did not complete the initial calibration session. Instead they were given a training block of 12 trials in which the probe was either the same as the final position or displaced 3 frames in either the predicted or unpredicted direction.

In the main experimental block, probes that were different from the final position were displaced by either 1 frame or 2 frames for all participants. The probe was therefore in one of five possible positions from the final position of the action sequence: 0 frames (the same position), 1 frame or 2 frames further along the observed trajectory (in a predicted position) or 1 frame or 2 frames in the opposite direction (in an unpredicted position).

The trial procedure was the same as in the main experiments. The joystick was placed to the right of the participant and oriented orthogonally to the direction of motion observed. Prior to action onset, participants pushed the joystick forward (away from them) if the object was safe to grasp, and pulled the joystick backward (toward them) if the object was painful to grasp. The executed actions were therefore orthogonal to the observed actions ensuring that any effects were not due to simple visual motion priming from observing one’s own actions. After action execution, the observed action sequence began. The SOA between onset of the executed action and onset of the observed action varied across the experiments by 1200 ms (Experiment 1) or 200 ms (Experiment 2). Participants then performed the representational momentum task by judging if a probe was in the same or different position as the final position of the action sequence. Each participant completed 180 trials composed of 3 repetitions of the factors Executed Action (pull, push), Observed Action (towards, away), Movie Length (3,4,5), and Probe (unpredicted[2], unpredicted[1], same[0], predicted[1], predicted[2]). Additionally, there were 16 catch trials in which the probe was displaced by 4 frames in either a predicted or unpredicted position and the movie length was always 5 frames.

**Results**

Participant exclusions (Experiment 1: n = 6, Experiment 2: n = 2) and trial exclusions (Experiment 1: 0.6%, Experiment 2: 0.8%) were based on the same criteria as in the main experiments. The results were analysed analogously to the main experiments. The different probe distances (1 frame, 2 frames) were collapsed to make a 3 level Probe factor (predicted, same, unpredicted).

The proportion of different responses were initially tested for the presence of an RM effect by conducting a mixed measured ANOVA with Probe as a repeated measures factor and Experimental Group (1200 ms SOA, 200 ms SOA) as an independent measures factor.

There was a significant effect of Probe (*F*(2,2) = 88.2, *p* < .001, *η_p_^2^* = .683). Probes in an unpredicted position elicited more different responses than probes in both a predicted position (*t*(42) = 7.13, *p* < .001, *d* = 1.29, *95% CI* [16,28]) or the same position as the final frame of the action sequence (*t*(42) = 13.4, *p* < .001, *d* = 2.2, *95% CI* [30,41]). Probes in the same position elicited less different responses than probes in a predicted position (*t*(42) = 6.39, *p* < .001, *d* = .79, *95% CI* [9,17]). There was no main effect of Experimental Group (*p* = .135) and no Interaction (*p* = .974).

The size of the RM effect, reflecting the increased detection of probes in an unpredicted position compared to probes in a predicted position (“different” responses for unpredicted probes minus predicted probes), was entered into a three way ANOVA with Executed Action Direction (Push, Pull) and Observed Action Direction (Toward, Away) as repeated measures factors, and Experimental Group as an independent measures factor.

The RM effect was larger after pushing the joystick than after pulling it (Executed Action: *F*(1,41) = 6.97, *p* = .012, *η_p_^2^* = .145), and this effect was significant only when the SOA was 200 ms (t(19) = 3.11, p = .006, d = .36, *95% CI* [3,14]) but not when it was 1200 ms (t(22) = .286, p = .778, d = .04, *95% CI* [-.05,.04]) (Executed Action*Experimental Group: *F*(1,41) = 4.55, *p* = .039, *η_p_^2^* = .100). Crucially, the RM effect was not influenced by the congruency of the Executed and Observed Actions (*F*(1,41) = 1.14, *p* = .292, *η_p_^2^* = .027) and there were no further main effects or interactions (all *F* < 1.51, *p* > .227).


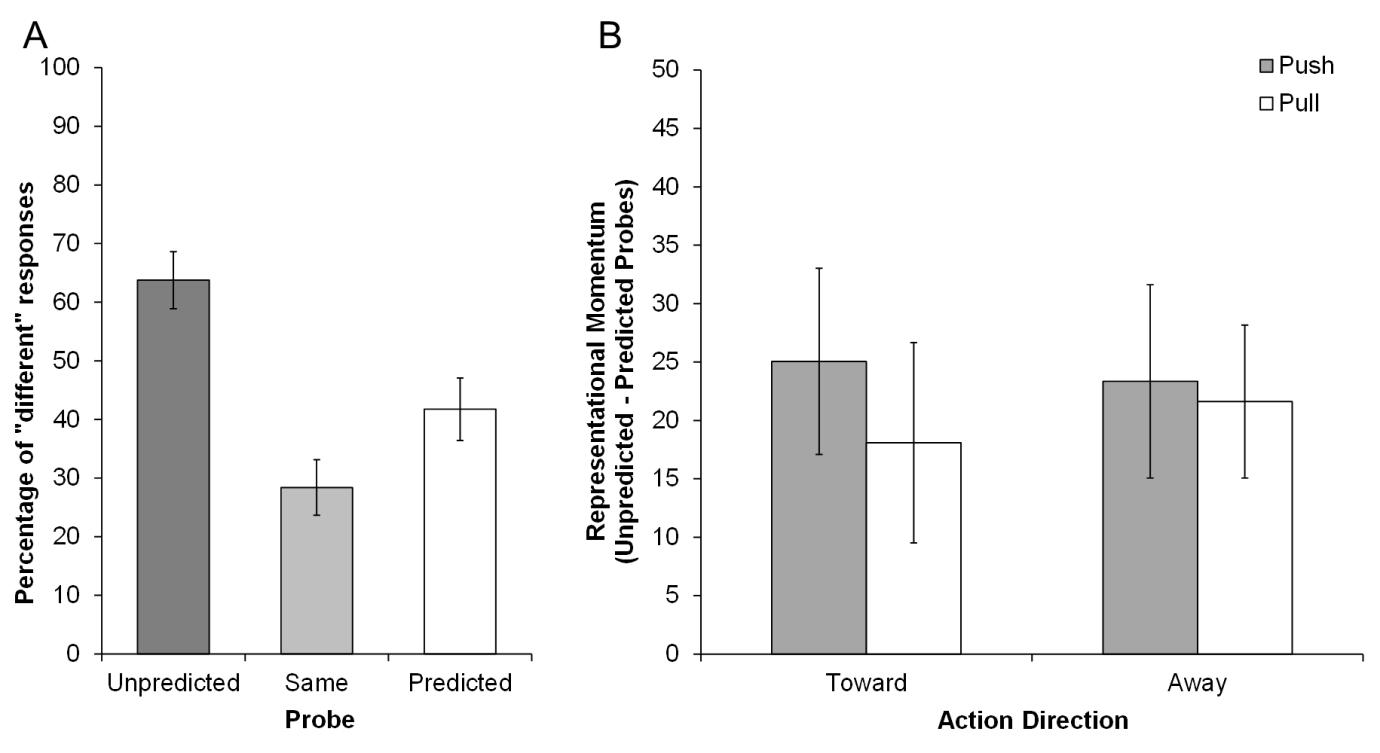


**Supplementary Fig. 4.**

The Representational Momentum effect and the effect of prior expectation. The proportion of reported displacements (a) when the probe was either the same as the final position of the action stimulus, or displaced in either a predicted or unpredicted position. The representational momentum effect (% “different” responses for unpredicted probes – predicted probes) (b) for each action direction after executing either a push or pull movement with the joystick. Error bars represent 95% confidence intervals.

**Discussion**

Despite a reliable RM effect being found, this was not influenced by the congruency of an action executed immediately prior to onset of the action. Predictive biases in social perception are therefore not caused by a general retrieval of the relevant action prior to observing it. Rather, it is critical that the observer generates an expectation of the actor’s forthcoming behaviour, as in the instruction situation used in the main experiments.
