# Supplementary material for: One Step Ahead: The Perceived Kinematics of Others’ Actions Are Biased Toward Expected Goals
Source: J Exp Psychol Gen. 2015 Nov 23;145(1):1–7. doi: 10.1037/xge0000126 (PMC4694084; doi:10.1037/xge0000126)
Supplement: Supplementary file 2 [file xge-XGE-2014-0717-Supplementary-Table.docx]

**Supplementary Table 1: Description of trials in Supplementary video**

All action sequences in the trial examples begin on frame 13 and are 5 frames long. The object type is always safe to grasp and the arm reaches toward the object. The first frame of the action sequence is on screen for 1000 ms, during which time the participant made their verbal response. The probe in trial 1 is the same (0) as the final frame of the action sequence, in the second trial it is backward by 4 frames, and in the third trial is forward of the final position by 4 frames. This three trial sequence is repeated 3 more times, each time reducing the distance of the different probes from the final position of the action sequence by 1 frame.
